# Subcyclo laser procedure results in patients with glaucoma

**Published:** 2018

**Authors:** Irina Lutic, Carmen Dragne, Mircea Filip, Andrei Filip, Miruna Nicolae, Raluca Moisescu, Ileana Ungureanu, Grigorios Triantafyllidis, Cristina Antonescu

**Affiliations:** * AMA Optimex Clinic, Bucharest, Romania

**Keywords:** subliminal cyclophotocoagulation, glaucoma, intraocular pressure

## Abstract

**Purpose.** To analyse the results of subCyclo (subliminal transscleral cyclophotocoagulation) laser procedure for patients with glaucoma.

**Material and methods.** The study included 50 eyes of 32 patients diagnosed with various subtypes of glaucoma for which we performed subliminal transscleral cyclophotocoagulation under retrobulbar anesthesia. After the procedure, all patients were advised to continue their antiglaucoma medication until further notice and we added a topical steroid for 2 weeks. The minimum follow-up period was 6 months.

**Results.** The mean IOP (intraocular pressure) decreased from the preoperative value of 26.27 mmHg (±6.52) to 15.9 mmHg (±5.72) one day after the treatment. At 7 days, the average IOP was 13.72 mmHg (±4.31), 15.81 mmHg (±3.69) at 6 weeks, 15.94 mmHg (±5.66) at 3 months and 16.32 mmHg (±5.24) at 6 months. The second intervention was performed in 9 eyes due to poor IOP control after the first procedure, two eyes developed ocular hypertonia 6-8 hours after the procedure and one eye presented mydriasis after the procedure.

**Conclusions.** Early results for this study showed that subCyclo laser procedure could determine a satisfactory decrease of the IOP with a low risk of complications.

**Abbreviations**:

IOP = intraocular pressure, TSCPC = transscleral cyclophotocoagulation, POAG = primary open-angle glaucoma, NVG = neovascular glaucoma, BCVA = best corrected visual acuity

## Introduction

Transscleral cyclophotocoagulation uses a continuous diode laser and it has been a treatment option in advanced glaucoma cases with suboptimal IOP control, for a long time. The main effect is the destruction of the ciliary body by acting on the following structures: ciliary epithelium and stroma. The main target of the laser is the melanin located in the pigmented ciliary body epithelium. The result of this treatment is diminished aqueous secretion. Because of its high rate of complications, TSCPC has been a last option in eyes with preserved function and uncontrolled, severe glaucoma when other treatment options are exhausted [**1**].

SubCyclo has the same principle of action as TSCPC but avoids the side effects of the previous mentioned procedure. It is a nondestructive laser treatment for all types of glaucoma, advanced or moderate stage, unresponsive to other therapeutic alternatives or in cases with high risk of surgery [**2**].

For this procedure, an infrared diode laser is used with 810 nm wavelength to stimulate the ciliary body structures and the uveoscleral pathway. This way it reduces the production of the aqueous humour and facilitates the uveoscleral outflow, with IOP reduction as a final effect.

The continuous-wave beam is segmented so that the whole energy is transmitted through ultrashort and repetitive pulses (they represent “time on”- when the laser is active) separated by rest periods (“time off”), which allow tissue cooling with minimal adjacent tissue damage. The main goal of our research was to analyse the profile of this procedure in terms of safety and efficacy of IOP reduction in glaucoma patients.

## Material and methods

In this research we included patients with various subtypes of glaucoma who underwent subliminal transscleral cyclophotocoagulation if they had worsening IOP control and/ or worsening visual fields, if they did not wish/ or were not good candidates for invasive surgeries.

The preoperative assessment included a complete ophthalmic evaluation.

The anesthesia consisted in a retrobulbar block of ropivacaine and 4% lidocaine delivered before the procedure.

The tip of the probe was moved in a ”painting” motion along the upper and lower hemisphere, 3 mm behind the limbus, sparing the 3 and 9 o'clock positions. The total time of the treatment was 160 seconds, corresponding to 80 seconds for each hemisphere.

We followed the recommendations of the producer and we adjusted the treatment settings as mentioned in **Table 1**.

**Table 1:** Treatment settings

Power level 2000mW **Duty Cycle 31.3%** (time on 0.5 sec, time off 1.1 sec)	Power level 2000mW **Duty Cycle 25%** (time on 0,62 sec, time off 1.9 sec)
• No previous history of severe ocular inflammation	• Active inflammation (conjunctival or uveal)
• No active inflammation (conjunctival or uveal)	• Preserved good BCVA or moderated visual field defects
• No severe keratitis or corneal ulcer	

Patients received a post-operative dose of prednisolone acetate ophthalmic suspension and a dose of levofloxacin hemihydrate and the eye was patched for 8 hours. All of them were started on topical steroid that was tapered progressively by the end of the second week after the laser and they were advised to continue the IOP-lowering treatment until further notice. Patients were seen postoperatively on day 1, day 7, week 6, months 3 and 6. At each check-up, we performed a slit-lamp examination, Goldmann tonometry and registered BCVA.

## Results

Fifty eyes of thirty-two patients with different types of glaucoma (summarized in **Table 2**) were accepted in this survey. There were 12 women and 20 men and the mean age was 54,7 years.

**Table 2:** Types of glaucoma

POAG	40 eyes
NVG	3 eyes
Uveitic glaucoma	2 eyes
Juvenile glaucoma	2 eyes
Pigmentary glaucoma	2 eyes
Posttraumatic glaucoma	1 eye

Baseline IOP was 26.27 mmHg (±6.52) and the average number of medications was 3.14. Mean IOP decreased to 15.9 mmHg (±5.72) one day after the procedure. At one week, mean IOP was 13.72 mmHg (±4.31), 15.81 mmHg (±3.69) at 6 weeks, 15.94 mmHg (±5.66) at 3 months. Six months after the laser, average IOP was 16.32 mmHg (±5.24) and the number of medications was 2,56.

**Fig. 1:**
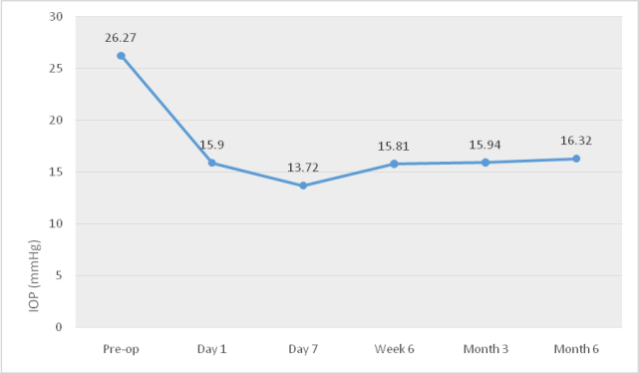
IOP evolution after subCyclo

For this period of follow-up, we registered a mean IOP reduction of 37% from baseline and 18.47% average reduction of medications (**Fig. 1**).

Two eyes developed ocular hypertonia 6-8 hours after the procedure, controlled with pressure-lowering medications, one eye presented mydriasis after the procedure, which responded to topical administration of Pilocarpine 1% for one week. Nine eyes required a second intervention due to inadequate IOP control after the first laser treatment (**Table 3**).

**Table 3:** Number of eyes requiring a second laser treatment

POAG	4 eyes
NVG	2 eyes
Uveitic glaucoma	1 eye
Juvenile glaucoma	1 eye
Pigmentary glaucoma	1 eye

No patient developed hypotony, all patients were pain free during and after the procedure and BCVA remained stable by the end of the follow-up.

## Discussions

Current glaucoma therapies include topical medications, laser therapies, microinvasive glaucoma surgery, and incisional glaucoma surgery. Subliminal transscleral cyclophotocoagulation does not induce fatal architectural damage to the cellular structure and proves to be an effective option to fill the gaps between topical medical therapy, MIGS, and traditional glaucoma-filtering surgery [**3**].

Evidence collected in the past years has proved that subliminal transscleral cyclophotocoagulation can provide a satisfactory decrease of IOP in patients with moderate to advanced glaucoma [**4**-**6**]. We must take into consideration the major advantages of this procedure: it has a reduced rate of complications and we can repeat it in order to improve the IOP control.

We must be aware that the location of the ciliary body varies between patients and even among quadrants of the same eye and adding transscleral illumination of the ciliary body can increase the accuracy of the probe localization.

In our opinion, we need more studies to optimize the profile of this treatment. In order to improve IOP control, we consider that the treatment settings must be adjusted to the type and the stage of glaucoma and to the type of the procedure (first intervention vs. reintervention).

Subliminal transscleral cyclophotocoagulation has a good profile in terms of safety and efficacy and this makes it a good option treatment for all cases of uncontrolled glaucoma, either as a single procedure or combined with glaucoma surgery [**7**].
